# Finding the Value in an Animal Health Data Economy: A Participatory Market Model Approach

**DOI:** 10.3389/fvets.2017.00145

**Published:** 2017-09-04

**Authors:** Patrick Lynch, Sinead Quealy

**Affiliations:** ^1^RIKON, Waterford Institute of Technology, Waterford, Ireland; ^2^Animal Disease Tracking Ireland Ltd., Kilmacthomas, Ireland

**Keywords:** animal health data economy, antimicrobial usage data collection, reframing data ownership, data tension, data farm aggregator

## Introduction

It is accepted that the costs of a severe animal disease outbreak in a country or region leads to significant economic costs, at a minimum in the tens of millions of euro (1). Increasing policy oversight of animal health by governments to prevent such disease outbreaks has increased the activity and growth of the animal health industry. A recent report by Grand View Research estimates that the animal health market can be valued at over €30 billion annually, growing at over 5% per annum (2). Yet, there is mounting consumer pressure on food retailers and processors to reduce the amount of animal medicines suspected of entering the food chain. This pressure stems from fears of potential ill effects on human health of, for example, growth hormones or antibiotics consumed by humans without their knowledge through contaminated food products. Governments and retailers are responding through tighter regulation on medicines usage and higher standards of food production on farm (3). In Europe, the EU Commission is currently streamlining the oversight of animal health through a new Animal Health Law, under the SANTE directorate general.

The new law encourages the use of technology and promotes near real-time surveillance and monitoring of drug usage and disease. These measures are to be welcomed but now must be implemented by Member States. Even a cursory glance at animal health agencies displays many researchers, projects, collaborative initiatives, and competent authorities with often duplicated roles, responsibilities, and geography. These myriad efforts share some common deficiencies—lack of usage data and lack of timely data (4). An issue that the lack of timely usage data in generating concerns within the broad animal health industry including veterinary and public health research is antimicrobial resistance (AMR). The gap in knowledge of antibiotic sales versus antibiotic usage on farms for food producing animals is a source of frustration for many. While in the EU, inspection, auditing, and data collection processes are in place, the lack of digitized, transferable, near real-time data and information prevents the animal health industry from meeting the AMR challenge as effectively as possible.

With the almost ubiquitous nature of smartphones in farming now, there are many solutions on the market to enhance the digitization of important usage data. However, making these data available to researchers and policy makers has often been blocked by a misrepresentation of farmers’ attitudes toward data ownership and a comfort with the *status quo* by agri-food and pharmaceutical industries. The most frequent roadblock erected in discussions around improved data capture and sharing is data ownership, specifically, “who owns the data?” This concept of “ownership” must be addressed, and we believe that emerging EU digital economy priorities enable a reframing of data ownership. This reframing will force the entire animal health industry to create fresh market and business models to meet existing and emerging needs of farmers, researchers, policy makers, and consumers.

## Data Tensions in the Animal Health Industry

At the heart of this big data tension between farmers and animal health technology providers (AHTP) is the issue of intellectual property rights and “who owns and controls the data.” In terms of ownership, the EU policy holds a clear delineation between data and information ownership; farmers own their farm data but when that raw data pass an inflection point where information is generated usually by an algorithm, there is a transition of data rights and ownership to the AHTP. From a farmer’s perspective, this policy position raises concerns around: (i) data access—who can see my data? And how is my data being used? (ii) data portability—do I have the flexibility to share and reuse data across interoperable applications? (iii) price discrimination—will service providers who also have farm data, tailor their prescribed solution and pricing based on farm attributes to maximize their profits from the farmer? On the other side, the AHTPs also have data concerns around protecting the intellectual property rights of their algorithms from competitors, especially if the farmer wants to work with a different AHTP in the future. While farmers would argue that they need to receive a fair share of the value generated from their data, AHTPs would counter claim that no one farmer’s data adds significant value to the margin, instead value is generated from their technical capability to aggregate data from many farms. However, given that there are only a limited number of farms in the EU (*circa* 10.8 million), the argument that autonomous animal health data of an individual farm has a near zero marginal value does not hold true. Moreover, the limited farm population base means that each customer acquisition has competitive consequences for AHTPs that adds real value to their profitability.

## Overcoming Data Tensions: Evolving to a Participatory Market Model

Although an emerging market place, the current animal health data market model can be classified as a single-side data economic model (5) where farmers are considered homogenous and exchanges follow a linear path as AHTPs extract data, transform it, and sell output. This type of market model is often labeled as captive prescriptive and is characterized by a closed business system mentality where there is low collaboration between value chain actors but high farmer engagement. In this market model, the farmer is a passive stakeholder of an integrated animal health data value chain and adopts the role of franchiser/contractor with limited freedom.

However, the importance attributed to farm data in animal health places farmers in a new context and redefines their role in the value chain. Farmers are becoming more aware of the value of their farm data and are moving from a passive value chain participant to becoming an integral, empowered, and participating stakeholder in the new data value chain. This participatory value chain adopts a network concept where actors collaborate to cocreate value that could not be created individually. In comparison to the traditional captive prescriptive market model, the complexity of this recharacterization of the animal health industry is significant as it will dramatically reshape the value model of the industry and the data-driven business models required by a participatory market model.

**Figure 1:**
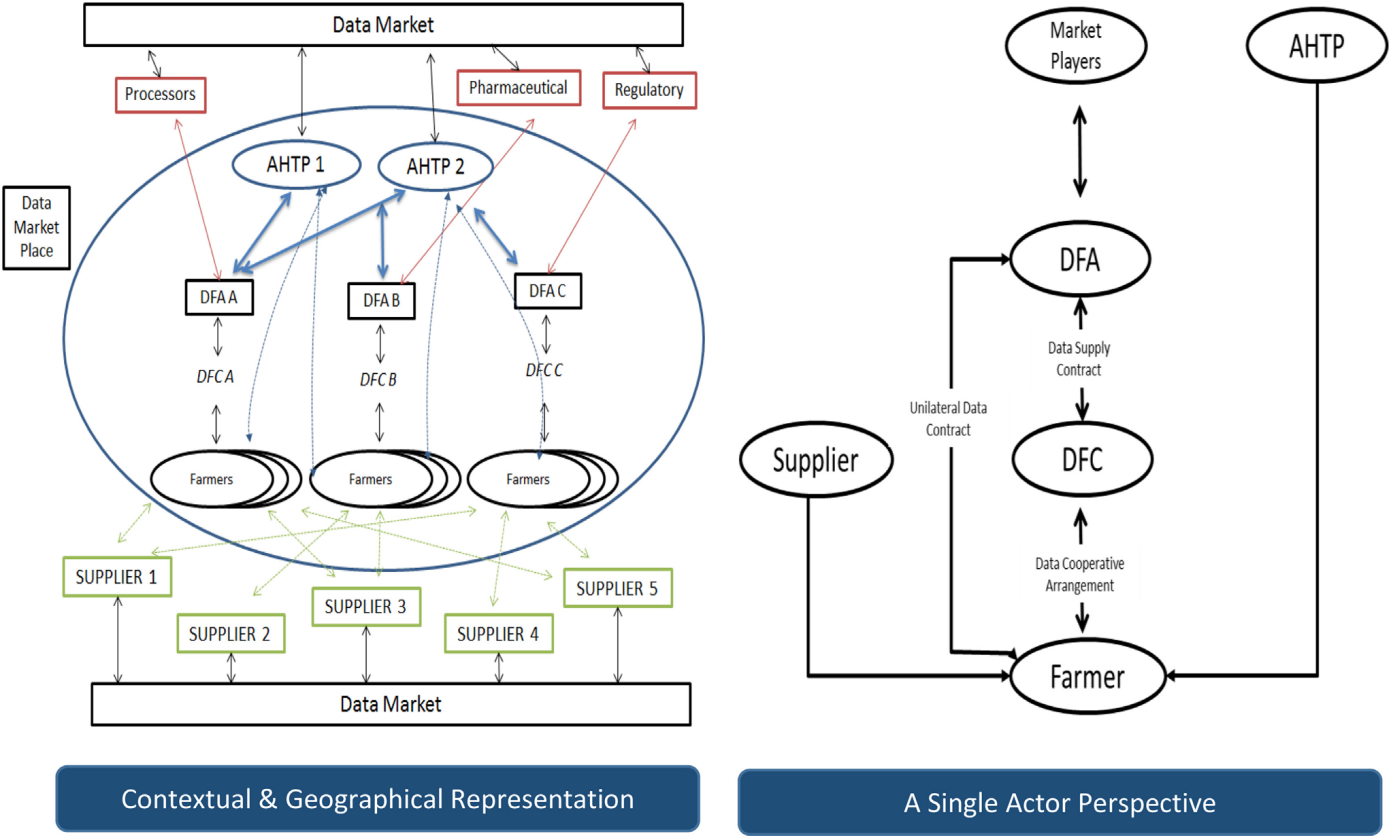
Participatory market model for animal health data.

Figure 1 below presents our conceptualization of a Participatory Market model for the Animal Health Industry from a contextual and geographical representation and from a single actor perspective. In this model, the farmer is an active participant in the animal health data value chain. The farmer adopts the role of data controller and manages how their data is used and shared for economic interest. AHTPs must seek permission to use the data for defined purposes or to share data with other third parties or to combine with other data and so on. By becoming an active value chain participant, the farmer benefits through accessing the data market place and using data to both enhance productivity gains, reduce disease or reputational risks to the industry and to get revenue from the provided data. Outside the Animal Health Industry, there are some promising examples of technologies and companies that promote farmers retaining control of their data. Datalinker is a DairyNZ project that has established a set of protocols that enable secure, standardized data interchange between organizations controlled by farmer permissions and electronic license agreements. Another example is Farmobile, a company that sells a data collection tool that centralizes growers’ agronomic data from multiple systems in one electronic farm record. Farmobile standardizes the data and makes it easily searchable for customers who want to purchase data and the farmers get 50% of the revenue derived from selling the data. These examples illustrate that to harness the data potential of individual farmers into useful quantities, aggregation is needed. What they also illustrate is that the participatory market place that is emerging resembles a platform business model where “*match-making*” intermediaries operate between AHTPs who need data as part of their value-added services and farmers who have adopted the role of data controller for economic benefit. In Figure 1, a new matchmaker role of a data farm aggregator (DFA) is foreseen in the animal health data value chain to act as an intermediator and facilitating agent between the Farmer and the AHTP market to exploit the active participation of the data farm community (DFC) to aggregate their data for the provision of commercial services to other market stakeholders while ensuring maximum value of the farmer’s data. In essence, the DFA is a broker between the different actors and is responsible for acquiring data from farmers, aggregating it into a portfolio and offering animal health data to different market players. In return, the DFA receives the value it creates from these markets and shares it with the farmers as an incentive to engage in the data market.

It is also envisaged that the farmer will become part of a DFC, which can be considered from two perspectives. First, the farmer is part of a DFC because they are contracted to the same DFA but have no knowledge of one another and are free to choose the DFA that they prefer. Participation in the DFC enables trading of aggregated data between the farmers in the DFC through the DFA. In the second perspective, the DFC is a self-organized entity or cooperative where farmers are a member and collaborate to ensure maximum returns on their combined data value generated from their data supply and services. Examples of DFCs can be considered to exist in some veterinary practices as discussion groups or health focus groups emerge to discuss breeding, bio-security, or other best practice. As can be seen from Figure 1, more than one DFA can operate within a geographical area. This type of participatory market design is referred to as a two-sided or multi-sided economic model because it acts as a matchmaker between different value chain stakeholders. Revenue models will be complex as different data transactions are answered and settled for. There will also be multiple types of buyers and/or sellers and, in fact, a single party can be both a buyer and a seller of data.

## Conclusion

Data is at the core of the emerging animal health market model landscape. Although embryonic and complex in nature, this new participatory market landscape holds significant new business opportunities. The emergence of the data farmer as a valid and integral value chain member means that companies must become more attuned to the needs of the farmer and design new value propositions to attract and secure their data contracts. Indeed, an acute challenge of participatory business models is that a critical mass of farmers will be required to be attractive as a data intermediary to the marketplace. This means that the DFA must devote much attention to designing innovative business strategies to get on-board as many early adopters as possible to drive this network effect. Considering no current actor in the traditional animal health industry has a participatory or multi-side nature to their business model, the complexity of this recharacterization, especially for incumbents will be significant. To date, animal health market actors have tended to focus and rely on technology innovation as the driving force for the evolution of the industry and this is within their comfort circle; however, the data-driven nature of these technology advancements will also require a call to action to engage in business model transformation which is new to most. What is being put forward here is that sophisticated new participatory business models are needed to support near real-time surveillance and monitoring of drug usage and disease with the additional benefit of making quality datasets available for research to meet animal and public health priorities, such as AMR.

## Author Contributions

Sinead Quealy provided the agricultural and animal health industry perspective, examples, and knowledge. Patrick Lynch applied his extensive work and expertise in market models and data-driven business models to the opportunities in agri-food and animal health industries to meet challenges in a shifting setting.

## Conflict of Interest Statement

The authors declare that the research was conducted in the absence of any commercial or financial relationships that could be construed as a potential conflict of interest. PL is the director of RIKON-WIT, a business innovation research centre located in Waterford Institute of Technology in Ireland. He has published extensively on market models, business models and networked innovation in top-tier journals and peer-reviewed conferences. He has amassed considerable industry, consultancy and applied innovation research experience in process optimisiation, business modelling & strategy and market models. He is the principal investigator on over 450 innovation and research projects that are recognised for making real transformational change in business and re-imaging how companies can identify tactical business opportunities to grow business volume. SQ is a cofounder and managing director of VirtualVet, a service developed to help farmers comply with EU legislation. VirtualVet digitizes on-farm animal health information on behalf of farmers and makes the information available for purchase by relevant organizations up along the value chain. During the last 2 years, VirtualVet has researched EU policy in the areas of agri-food and animal health, barriers to farmer adoption of technology, and the emergence of data-driven business models around the world. This experience informs the opinions in this article.
